# Comparison of the value of the GI-RADS and ADNEX models in the diagnosis of adnexal tumors by junior physicians

**DOI:** 10.3389/fonc.2024.1435636

**Published:** 2024-08-16

**Authors:** Yongjian Chen, Yanru Li, Huiling Su, Guorong Lyu

**Affiliations:** ^1^ Department of Ultrasound, the Second Affiliated Hospital of Fujian Medical University, Quanzhou, China; ^2^ School of Clinical Medicine, Quanzhou Medical College, Quanzhou, China

**Keywords:** ovary, ultrasonography, tumor, GI-RADS, ADNEX model

## Abstract

**Objective:**

To compare the diagnostic effectiveness of the Gynecologic Imaging Reporting and Data System (GI-RADS) and Neoplasias in the Adnexa (ADNEX) model for the diagnosis of benign and malignant ovarian tumors by junior physicians.

**Methods:**

The sonographic data of 634 patients with ovarian tumors confirmed by pathology in our hospital over 4 years were analyzed retrospectively by junior doctors. The diagnostic efficacy of the GI-RADS and ADNEX models was compared based on pathology.

**Results:**

(1) Regarding the diagnostic efficacy of the GI-RADS and ADNEX models, the sensitivity was 90.15% and 84.85%, the specificity was 87.65% and 85.86%, the accuracy rates were 88.17% and 85.65%, and the Youden Indices were 0.778 and 0.707, respectively. The areas under the receiver operating characteristic (ROC) curves were 0.924 (95% CI: 0.900-0.943) and 0.933 (95% CI: 0.911-0.951), respectively. The GI-RADS classification was equivalent to that of the ADNEX model in the diagnosis of adnexal tumors (*P>*0.05). These findings were highly consistent with the pathological results (Kappa values were 0.684 and 0.691, respectively). (2) When differentiating between different pathological types of adnexal tumors, the ADNEX model had the best diagnostic value for distinguishing between benign tumors and stage II-IV ovarian cancer (AUC=0.990, 95% CI: 0.978-0.997).

**Conclusions:**

(1) The diagnostic efficacy of the GI-RADS and ADNEX models in the diagnosis of benign and malignant ovarian tumors by junior physicians is excellent and comparable. (2) The ADNEX model shows good value for differentiating ovarian tumors of different pathological types by junior physicians.

## Introduction

Ovarian malignancy is one of the deadliest and most complex diseases in women, and early diagnosis is difficult. With early detection and intervention, the 5-year survival rate can reach 90% (1). Ultrasonography is one of the methods for the early diagnosis of adnexal masses. There are many risk stratification models for the ultrasound diagnosis of ovarian tumors. The most commonly used models are the ovarian Imaging Reporting and Data System (O-RADS), gynecological imaging reporting and data system (GI-RADS), simple rule (SR) and ADNEX models. Among these models, O-RADS is a new model proposed by the American College of Radiology for the diagnosis of ovarian tumors. The GI-RADS and IOTA simple rules models are qualitative diagnostic models (2, 3) with greater flexibility, while the ADNEX model is a quantitative procedural diagnostic model and a model that can distinguish borderline tumors. Therefore, this paper chooses qualitative and quantitative procedural models for comparison. However, there are few reports on the comparison of diagnostic performance between the GI-RADS and ADNEX models. And two related studies found that GI-RADS and ADNEX showed little difference in diagnostic performance (4, 5). In addition, the subjective assessment of ultrasound experts is still the best method for the preoperative identification of adnexal tumors (6, 7), but due to the lack of ultrasound expert resources, ultrasound identification of ovarian tumors is still performed by junior doctors in developing and underdeveloped country in most cases (4, 5). Therefore, this study aimed to explore the value of the GI-RADS classification system and the ADNEX model in the diagnosis of ovarian tumors by junior specialists.

## Materials and methods

### Participants

The clinical data of 634 patients with ovarian tumors who underwent surgical resection in our hospital during a 4-year period were retrospectively collected. All patients underwent preoperative ultrasound examination, and pathological results were obtained. The mean age was 39.59 ± 13.28 years (range, 15-82 years). There were 499 premenopausal patients and 135 postmenopausal patients. There were 502 patients with benign masses, 35 patients with borderline masses and 97 patients with malignant masses.

The inclusion criteria were as follows: ① age over 14 years; ② definite postoperative pathological staging; ③ saved sonograms were clear and complete, and the report writing was standardized; and ④ serum cancer antigen 125 (CA125) was detected one week before the operation.

The exclusion criteria were as follows: ① incomplete clinical or pathological data; and ② previous ovarian tumor surgery or drug treatment.

This study was approved by the Ethics Committee of the Second Affiliated Hospital of Fujian Medical University (Ethics No. 2022519).

### Instruments and methods

#### Ultrasonic inspection

GE Voluson E10 (General Electric, USA), GE Voluson E8 (General Electric, USA) and other color Doppler ultrasound diagnostic instruments were used. The transabdominal ultrasound probe was operated at 3.5-6.5 MHz, and the transluminal ultrasound probe was operated at 5-9 MHz. The images and ultrasound reports were stored in a standardized manner. When there was more than one lesion in the adnexal region on ultrasound examination, the lesion with the most complex ultrasound appearance or the lesion with the largest mass was selected when the ultrasound appearance was similar, and the results were evaluated. And junior doctors analyzed the ultrasound images afterward.

#### GI-RADS classification and ADNEX model judgement method

The ADNEX model consists of six ultrasound indicators and three clinical indicators. By inputting the required indicators into the ADNEX model, the nature and staging results of adnexal masses can be obtained (8). A risk of an ovarian mass ≥10% was considered malignant. The absolute risk value was used to calculate the subtype of malignant tumor, and the subtype evaluation results included benign, stage I, II-IV, metastatic cancer, and borderline cancer. For GI-RADS classification, 634 images were numbered and sorted by one doctor and then randomly assigned by a computer to another junior doctor (with 5 years of work experience) who was unaware of the diagnosis of the images. The GI-RADS5 classification method was used to classify GI-RADS categories 1 to 3 as benign lesions and categories 4a to 5 as malignant lesions (3).

### Ease of use of GI-RADS classification and ADNEX model

The questionnaire was conducted regarding ease of use of GI-RADS classification and ADNEX model. The inclusion criteria were that the participants were Junior physicians with less than three years of service. Data collection took place across 5 main hospitals in mainland China: the Second Affiliated Hospital of Fujian Medical University, Quanzhou First Hospital, Zhangzhou Hospital, the First Affiliated Hospital of Anhui Medical University, and Quanzhou women’s and children’s hospital. The response choices were presented using a 4-point Likert scale: 4=strongly agree, 3=agree, 2=disagree, and 1=strongly disagree.

### Statistical analysis

SPSS 20.0 software was used for statistical analysis. The count data are expressed as frequencies and rates, and the diagnostic efficacy of ultrasound GI-RADS classification and the ADNEX model were calculated (the borderline tumors were classified as malignant). The consistency of the GI-RADS classification, ADNEX model and pathological results was tested, and the kappa value was calculated. The sensitivity, specificity and accuracy of the GI-RADS classification system and the ADNEX model were compared via the McNemar test. The receiver operating characteristic (ROC) curve was drawn, the area under the curve (AUC) was calculated, and the Delong test was used for comparison.

## Results

### The GI-RADS classification and ADNEX model were used to determine the results of the included ovarian tumors

The pathological diagnosis, GI-RADS classification and ADNEX model results for the adnexal tumors included in the study are shown in **Table 1**.

**Table 1:** Pathological diagnosis of various accessory tumors, GI-RADS judgement results and ADNEX model judgement results.

pathology results	GI-RADS		ADNEX		
	1-3categories	4a-5categories	<10%	≥10%	Total
ovarian serous cystadenoma	70	2	70	2	72
ovarian mucious cystadenoma	68	8	71	5	76
ovarian seromucinous cystadenoma	6	2	8	0	8
ovarian adenfibroma	6	7	11	2	13
ovarian fibroma	0	11	4	7	11
ovarian theca cell tumor	0	2	0	2	2
ovarian lipoma	1	0	1	0	1
struma ovary	3	2	3	2	5
ovarian simple cyst	8	0	8	0	8
paroophoritic cyst	5	0	5	0	5
ovarian luteal cyst	1	2	3	0	3
ovarian luteinized follicular cyst	1	0	1	0	1
ovarian follicular membrane cyst	1	0	1	0	1
hydrosalpinx	15	2	16	1	17
ovarian endometrioid cyst	143	12	151	4	155
ovarian mature cystic teratoma	112	12	78	46	124
ovarian clear cell carcinoma	0	11	0	11	11
ovarian serous carcinoma	2	40	1	41	42
ovarian mucinous cancer	1	7	2	6	8
ovarian endometrioid carcinoma	1	7	0	8	8
ovarian mucinous cancer	0	3	0	3	3
ovarian granulose cell tumor	2	7	3	6	9
Ovarian endometrial stromal sarcoma	0	2	1	1	2
ovarian asexual cell tumor	0	4	0	4	4
ovarian immature teratoma	0	3	0	3	3
ovarian metastatic carcinoma	0	7	0	7	7
ovarian borderline serous tumor	2	16	6	12	18
ovarian borderline mucous tumor	5	10	7	8	15
ovarian junctional endometrioid tumor	0	2	0	2	2

### Reliability analysis

The kappa indexes of GI-RADS and ADNEX were 0.684 and 0.619, respectively.

### Comparison of the diagnostic efficacy of the GI-RADS classification and the ADNEX model

**Figure 1:**
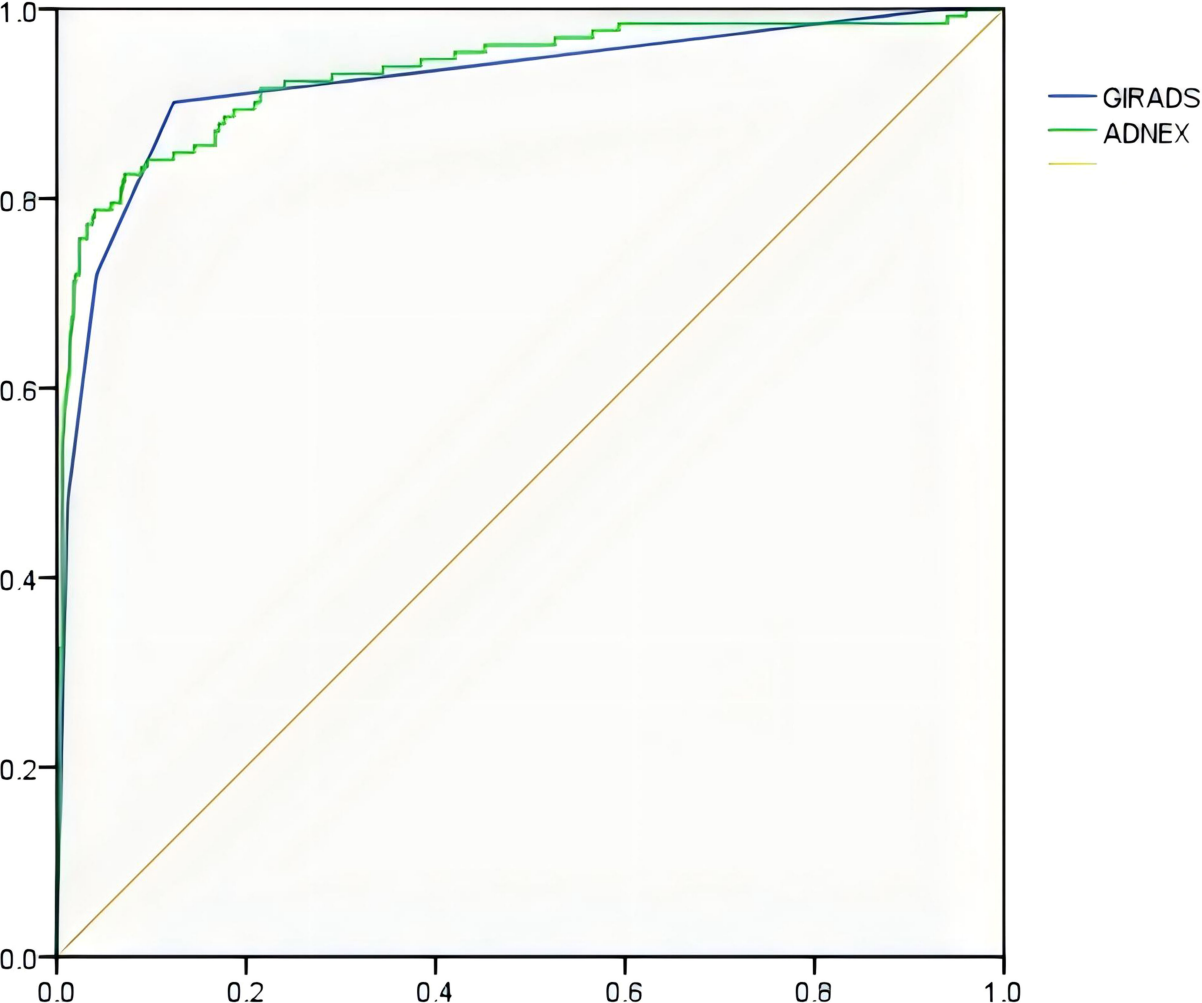
ROC curve of the GI-RADS and ADNEX models for identifying accessory benign and malignant tumors.

**Figure 2:**
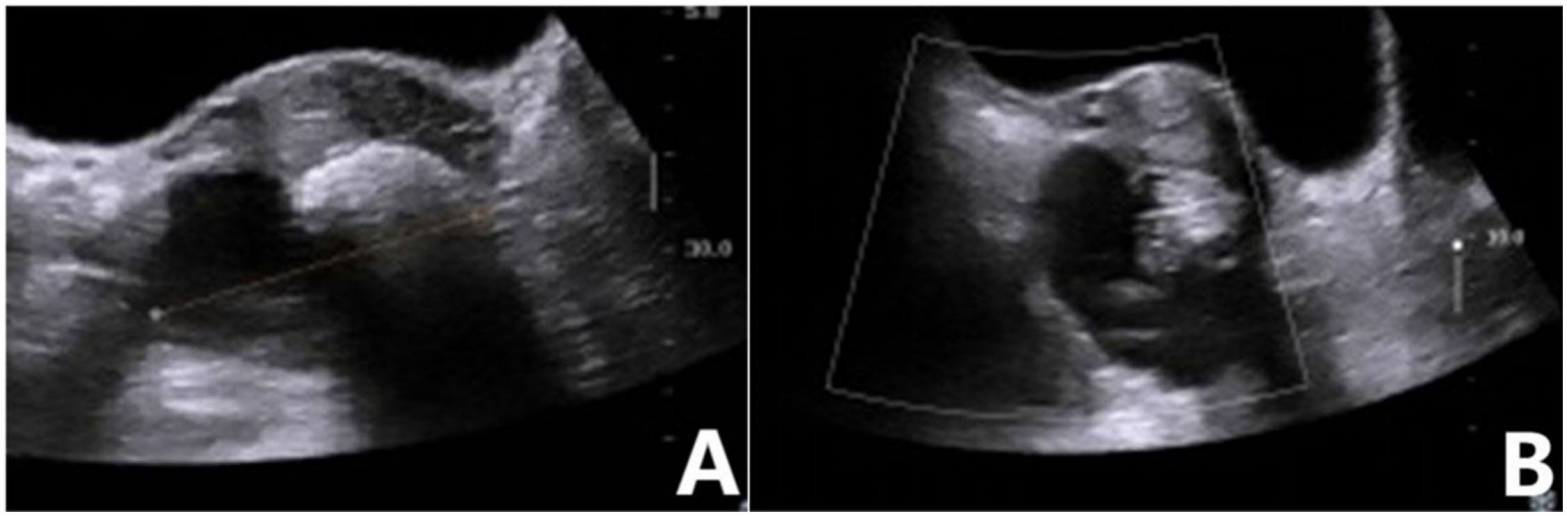
Mature teratoma of the ovary. **(A)** Two-dimensional ultrasound showed a mixed echo in the right adnexa, with a mass of strong echo and an acoustic shadow behind it. **(B)** No blood flow signal was detected in the mass. GI-RADS classification was as follows: category 3, mature teratoma diagnosed by ultrasound. The risk value of the ADNEX model was 1.8%, indicating that the lesion was a benign mass. Postoperative pathological revealed a mature teratoma.

**Figure 3:**
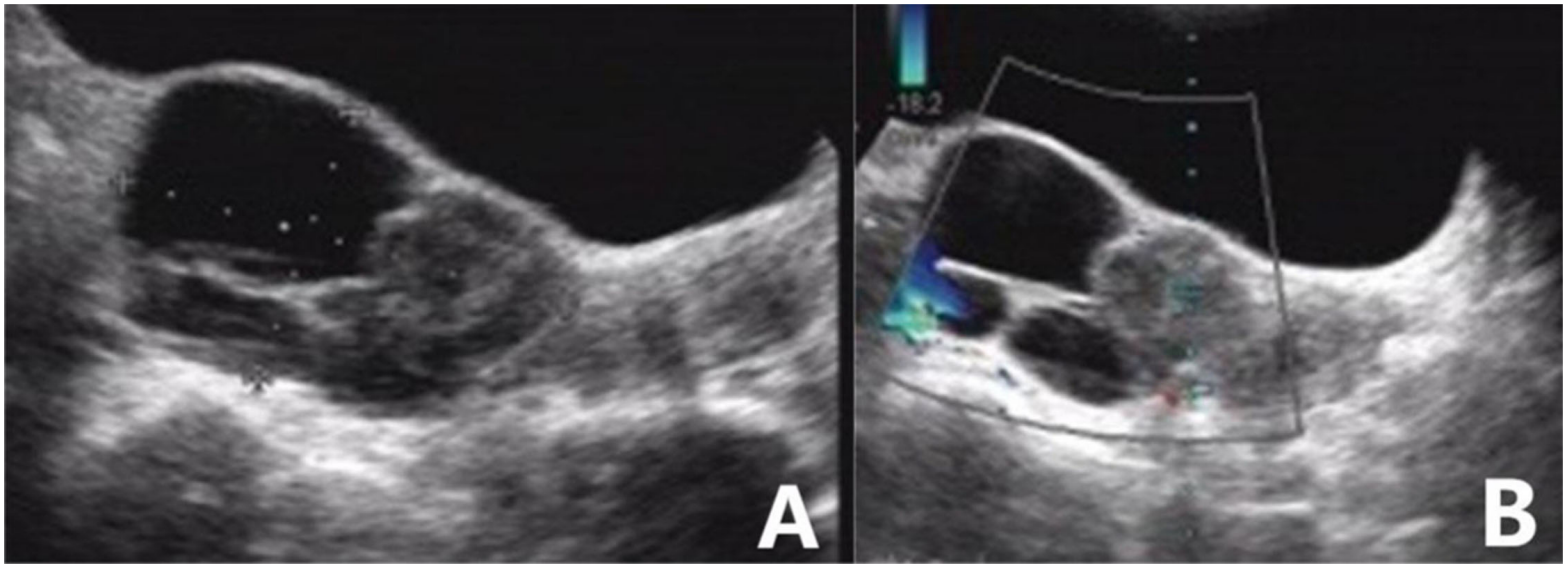
High-grade serous carcinoma of the ovary. **(A)** Sonography revealed a mixed echogenic mass in the posterior right of the uterus, with well-circumscribed cysts, a solid half, and internal multilocular septa. **(B)** A small amount of blood flow signal could be detected in the solid component of the mass. The blood flow parameters were as follows: PSV, 12.5 cm/s; EDV, 4.2 cm/s; and RI, 0.67. The GI-RADS classification was 4a, and the tumor was considered to be malignant. The risk value of the ADNEX model was 57.8%; for malignant tumors, the pathological type was predicted to be stage II-IV epithelial ovarian cancer. Postoperative pathological results revealed high-grade serous carcinoma.

The diagnostic performance of the GI-RADS hierarchical model was similar to that of the ADNEX model (all *P* > 0.05). For details, see **Table 2** and **Figure 1**. The kappa values were 0.684 and 0.619, respectively (**Figures 2**, **3**).

**Table 2:** Comparison of diagnostic efficiency between the GI-RADS and ADNEX models.

	Sensibility	Specificity	Positive predictive value	Negative predictive value	Accuracy rate	Youden index	AUC(95%CI)
GI-RADS	90.15%	87.65%	65.75%	97.13%	88.17%	0.778	0.924(0.900∼0.943)
ADNEX	84.85%	85.86%	61.20%	95.57%	85.65%	0.707	0.933(0.911∼0.951)

### Efficacy of the ADNEX model in identifying adnexal tumors

The efficacy of the ADNEX model in identifying 634 adnexal tumors is shown in **Table 3**. **Table 3** shows that the ADNEX model had the best ability to differentiate between benign masses and stage II-IV ovarian cancer, with an AUC of 0.990. The AUC was 0.689 for distinguishing stage I ovarian cancer from metastatic cancer and 0.735 for distinguishing stage I ovarian cancer from borderline tumors.

**Table 3:** The ADNEX model distinguishes the AUCs of different pathological attachment tumors.

Mass type	AUC (95%CI)
benign and borderline	0.891 (0.861∼0.916)
benign and stage I	0.926 (0.901∼0.947)
benign and stageII-IV	0.990 (0.978∼0.997)
benign and metastatic cancers	0.892 (0.862∼0.917)
borderline and stage I	0.735 (0.620∼0.830)
borderline and stage II-IV	0.925 (0.847∼0.971)
borderline and metastatic cancer	0.890 (0.755∼0.965)
stage I and stage II-IV	0.856 (0.766∼0.921)
stage I and metastatic cancer	0.689 (0.538∼0.816)
stage II-IV and metastatic cancer	0.886 (0.774∼0.955)

### Ease of use of GI-RADS classification and ADNEX model

**Table 4** shows the proportion of responses on individual items of perceived ease of use of GI-RADS classification and ADNEX model.

**Table 4:** Responses regarding perceived ease of use of GI-RADS and ADNEX model (N=102).

Items	Strongly agree, n (%)	Agree, n (%)	Disagree, n (%)	Strongly disagree, n (%)
The GI-RADS is convenient and easy to use	59 (57.8)	38 (37.3)	3 (2.9)	2 (2.0)
The ADNEX model is convenient and easy to use	49 (48.0)	37 (36.3)	10 (9.8)	6 (5.9)

## Discussion

In recent years, the methods and levels of systemic treatment for ovarian cancer have significantly improved, but due to the lack of effective early detection strategies, more than 70% of patients are in the advanced stage, and their 5-year survival rate has not significantly improved (9). Accurate prediction of the type of ovarian tumor is crucial in treatment decisions. The treatment of different types of ovarian tumors is very different. For benign adnexal masses, conservative treatment or less invasive surgery can be selected, and for suspected malignant masses, immediate referral to a gynecologic oncologist for appropriate staging and more extensive surgery is required (10, 11). Accurate prediction can not only save the lives of patients with ovarian cancer but also reduce the cost of treatment. Therefore, early and accurate diagnosis is particularly important.

There are many examination methods for the diagnosis of ovarian tumors, such as tumor-related markers, CT, MRI, and ultrasound. Ultrasonography is the first choice for the diagnosis of gynecological tumors and plays a very important role in the early evaluation of adnexal masses. Studies have shown (6, 12) that subjective assessment by ultrasound experts is an effective method for differentiating benign and malignant adnexal masses with an accuracy of 91%. However, due to the lack of ultrasound experts, ultrasound identification of ovarian masses is mostly performed by junior doctors. Due to the limitations of ultrasound examination experience, it is difficult for these junior doctors to accurately diagnose benign and malignant ovarian tumors before surgery. Therefore, it is necessary to find a simple ultrasound diagnostic model for ovarian tumors that is suitable for sonographers with limited clinical experience.

The GI-RADS classification was similar to the BI-RADS classification, that is, category 1: normal attachment; Category 2: positive benign lesions, generally considered the original functional lesions included in this category, such as the corpus luteum or simple ovarian cysts; Category 3: possible benign lesions, that is, large nonneoplastic ovarian tumors or teratomas, chocolate cysts, hydrosalpinx, etc.; Category 4: suspicious malignancy, that is, there is more than one malignant ultrasound indicator; Category 5: possible malignancy, that is, more than 3 malignant ultrasound indicators; Category 6: definite malignancy (confirmed by pathology). Malignant indicators included the following: (1) large papillary protrusion (≥7 mm); (2) solid or predominantly solid lesions (cystic portion < 50%); (3) thick septum (≥3 mm); (4) central flow; (5) resistance index (RI) < 0.50; and (6) complications with ascites. The ADNEX model included 3 clinical indicators (age (years), serum CA125 level, diagnosis and treatment in the gynecological tumor center) and 6 ultrasound imaging indicators (the maximum diameter of the tumor (mm), the maximum diameter of the solid component (mm), the number of papillary processes, whether more than 10 compartments, ascites, and whether there was an acoustic shadow behind the mass). The former is judged by the doctor, and the latter is judged by the doctor according to the ultrasonographic performance input to the computer. There are few reports on the comparison between these two models (4, 5, 13).

The diagnostic value of GI-RADS classification system and the ADNEX model for adnexal tumors by junior radiologists is rarely discussed in current studies. Therefore, this study aimed to compare them directly. The results showed that both methods can distinguish benign and malignant adnexal tumors, the qualitative diagnosis of adnexal tumors is highly consistent with the pathological diagnosis, and the diagnostic efficiency is better. Therefore, it can be used according to the clinical situation and personal preference, such as whether it is a tumor center or whether it has the ability to perform CA125 detection and ovarian tumor differentiation.

Compared with the relevant studies of Chen and Li et al. (3, 14), the diagnostic efficacy of the GI-RADS classification in this study for distinguishing benign and malignant adnexal tumors was lower for the following reasons: ① The GI-RADS classification should be based on the experience of the inspector to exclude endometrioid cysts, hydrosalpinx and other characteristic benign masses (15). However, in this study, the analysis was carried out by junior doctors with a lack of experience, and the diagnostic accuracy was lower than that of experienced ultrasound doctors. ② In this study, benign masses accounted for 79.18%, malignant masses accounted for 20.82%, the proportion of malignant masses was significantly greater than that in the literature (3), and the proportion of borderline and stage I masses among malignant masses was high, which caused difficulties in diagnosis.

In our study, the diagnostic performance of the ADNEX model was similar to that of the Araujo and Meys models. Araujo et al. (16) used the ADNEX model to differentiate benign and malignant ovarian tumors, and the results showed that the AUC was 0.925. The results of Meys et al. (13) showed that the AUC was 0.930. However, compared with the study of Meys et al. (13), the sensitivity of the ADNEX model in this study was significantly lower, while the specificity was greater. According to the analysis of the included subjects, the ratio of benign to malignant tumors was significantly greater than that reported by Meys et al. (13) (3.80 vs. 1.83). The proportions of patients with borderline and stage I tumors were also greater among those with malignant tumors (0.57 vs. 0.39).

The ADNEX model is the first risk model to distinguish between the four subtypes of benign, borderline, malignant, and metastatic adnexal masses. The results of this study showed that the model had good performance in distinguishing benign masses from borderline, metastatic and malignant masses (AUC between 0.891 and 0.990). In addition, the model was able to distinguish stage II-IV ovarian cancer from other malignant masses (i.e., borderline mass, stage I ovarian cancer, and metastatic cancer) (the area under the curve (AUC) ranged from 0.856 to 0.925). However, this model performs poorly in distinguishing stage I ovarian cancer from metastatic cancer and stage I ovarian cancer from borderline masses, which is consistent with the results of Araujo et al. (16). Thus, the diagnosis and differential diagnosis of borderline masses, stage I ovarian cancer, and metastatic cancer remain challenging in ultrasound prediction models. Therefore, attention should be given to comprehensive multidisciplinary clinical evaluations combined with MRI and contrast-enhanced ultrasound when necessary. In conclusion, our findings demonstrate the value of the ADNEX model used by junior physicians in the identification of different types of adnexal masses. The use of this model may lead to more accurate classification of cases, help prioritize patient surgical and treatment decisions, add diagnostic information to cases with indications for minimally invasive surgery, and optimize the management of patients with adnexal masses.

The results of our questionnaire showed that the ease of use of GI-RADS was superior to that of the ADNEX model among junior physicians. This may be due to the fact that the use of ADNEX model requires formulas or built-in applications for calculation, and its clinical application is limited to a certain extent. However, it is worth emphasizing that the ADNEX model is still simple to use, but it is more complicated to use than GI-RADS. Our results also show that The kappa indexes of GI-RADS and ADNEX were substantial. This means that the two not only have a certain ease of use, the decision results are in good consistency with the pathological results.

Compared with the relevant studies (4, 5), the advantage of this study is that our study compared the diagnostic efficacy of GI-RADS and ADNEX used by junior physicians to diagnose ovarian masses and found that both were equally effective. Lai, and others study did not compare to them. In addition, this study further confirmed the good efficacy of ADNEX in the differential diagnosis of benign, malignant, borderline and different stages of malignant tumors (AUC between 0.856 and 0.926) and poor efficacy of ADNEX in distinguishing stage I ovarian cancer from metastatic cancer and stage I ovarian cancer from borderline masses (AUC between 0.689 and 0.735). However, there are still some shortcomings in this study. First, the type of ultrasound instrument used is heterogeneous. However, it should be considered that two diagnostic tools must be applicable in clinical practice in any setting. Second, this was a single-center study.

In conclusion, both the GI-RADS classification and the ADNEX model can be used by junior sonologists to diagnose and differentiate ovarian tumors, and the ADNEX model can also distinguish different types of adnexal tumors. The GI-RADS classification does not need to be calculated by software, which is relatively simple and feasible and is more conducive to the screening of malignant adnexal tumors. The ADNEX model can be used to distinguish different types of adnexal tumors.

## Data Availability

The original contributions presented in the study are included in the article/supplementary material. Further inquiries can be directed to the corresponding author.
